# Lateral approach in robotic total knee arthroplasty for valgus knees: A step-by-step technique

**DOI:** 10.1051/sicotj/2025017

**Published:** 2025-03-27

**Authors:** Luca Andriollo, Pietro Gregori, Christos Koutserimpas, Elvire Servien, Cécile Batailler, Pascal Kouyoumdjian, Sébastien Lustig

**Affiliations:** 1 Orthopaedics Surgery and Sports Medicine Department, FIFA Medical Center of Excellence, Croix-Rousse Hospital, Hospices Civils de Lyon, Lyon North University Hospital 103 Grande Rue de la Croix-Rousse 69004 Lyon France; 2 LIBM-EA 7424, Interuniversity Laboratory of Biology of Mobility, Claude Bernard Lyon 1 University 69100 Lyon France; 3 Univ Lyon, Claude Bernard Lyon 1 University, IFSTTAR, LBMC UMR_T9406 69622 Lyon France; 4 Orthopedic and Traumatology Surgery Department, CHU Nîmes, University Montpellier 1 Nîmes, Place du Professeur Robert Debré 30029 Nîmes France; 5 Laboratory LMGC, CNRS UMR 5508, University of Montpellier II 860 Rue de St – Priest 34090 Montpellier France; 6 Laboratoire de Mécanique et Génie Civile (LMGC), UM 5508 CNRS-UM1 860 Rue de St – Priest 34090 Montpellier France

**Keywords:** Personalized knee arthroplasty, Robotic knee, Functional knee positioning, Functional alignment, Valgus knee

## Abstract

Total knee arthroplasty (TKA) in valgus knee deformities presents unique challenges, including alignment, soft tissue balance, and implant positioning. The lateral approach offers advantages over the traditional medial approach by improving direct access, patellar tracking, and soft tissue preservation. Robotic-assisted TKA enhances precision, ligament balancing, and patient-specific alignment strategies, such as functional knee positioning (FKP). This study describes a surgical technique integrating the lateral approach with robotic-assisted TKA using FKP principles. The technique is based on an image-based robotic system, ensuring accurate preoperative planning, intraoperative adjustments, and optimized prosthetic placement. Key intraoperative steps, including bone resection strategies, soft tissue balancing, and trial component evaluations, are detailed. The lateral robotic approach with FKP was found to be effective and reproducible, allowing for precise implant alignment and optimized soft tissue balance in valgus knees. This method minimizes the need for extensive lateral releases, preserves vascularity, and ensures postoperative stability. The combination of the lateral approach, robotic-assisted TKA, and FKP represents a promising strategy for valgus knee deformities. Further long-term studies are needed to validate the durability and functional benefits of this technique.

## Introduction

Total knee arthroplasty (TKA) in valgus knees presents challenges in alignment, soft tissue balance, and implant positioning, making the traditional medial approach less effective due to limited posterolateral access and the need for extensive lateral release [1]. In fact, valgus knee deformity is known to pose a significant challenge in TKA due to its impact on bone remodeling and the contraction or lengthening of soft tissues [2].

The lateral approach provides direct exposure, improves soft tissue balance, enhances patellar tracking, and preserves vascular integrity to the quadriceps-patellar tendon and lateral skin [3]. Table 1 presents the main advantages and limitations of the medial and lateral approaches [4].

**Table 1:** Comparison of the medial and lateral approaches for TKA in valgus knee deformity postoperative [TKA: total knee arthroplasty; KSS: Knee Society Score; ROM: range of motion; IT: iliotibial].

Approach	Advantages	Limitations
Medial	More familiar to most surgeons Easier and quicker exposure in standard TKA cases Higher postoperative KSS and ROM (though differences are minimal) Comparable surgical time to the lateral approach Reliable correction of valgus deformity	Requires extensive medial release, which may lead to instability Risk of overcorrection or residual valgus Less effective in restoring the anatomical axis in some cases (accurate restoration only in 22–30%) Possible increased risk of peroneal nerve palsy
Lateral	Better access to contracted lateral structures (IT band, posterolateral capsule) More anatomical restoration of knee alignment Less risk of overcorrection or medial instability Avoids excessive medial release	Technically more challenging Longer learning curve for surgeons Higher risk of patellar tracking issues May require tibial tubercle osteotomy or quadriceps snip for exposure in severe valgus knees Higher rates of intraoperative fractures and patellar complications

Additionally, robotic-assisted TKA offers greater accuracy in bone resection and ligament balancing, optimizing outcomes in complex knee morphotypes and enabling the implementation of personalized alignment concepts, such as functional knee positioning (FKP) [5–7]. The main challenge is that most robotic-assisted technologies have been developed specifically for the medial approach to the knee. Currently, no data is available on integrating robotic-assisted technology for valgus knees using a lateral approach.

This surgical technique aims to describe the integration of the lateral approach with robotic-assisted TKA in valgus knee deformities, utilizing an image-based robotic system (Mako^®^, Stryker^®^, Mahwah, USA).

## Surgical technique

**Figure 1:**
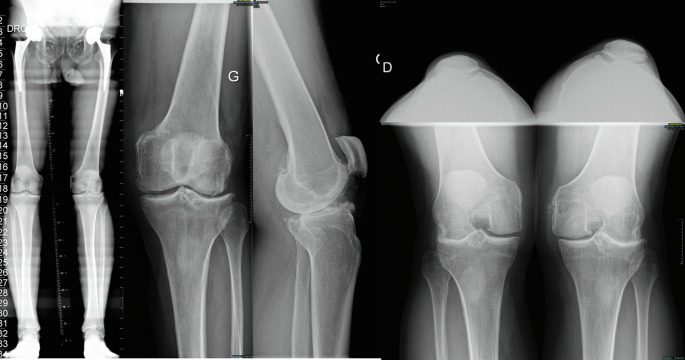
Preoperative x-rays of a valgus knee with osteoarthritis treated with image-based total knee arthroplasty following the principles of functional knee positioning, using a lateral approach. The case is presented in Video 1.

The surgical technique is shown in Video 1. Figure 1 illustrates the preoperative x-rays.

The patient is placed supine, one arm on a lateral support and the other on the table. A lateral and distal pad maintain 90° knee flexion. Without a tourniquet, a lateral skin incision is made.

### Step 1: Pre-operative planning for distal femoral & tibial plateau resection

Pre-operative planning involves determining the positioning of the prosthetic implant using a specialized computer navigation system (Orthomap ASM^®^, Stryker^®^, Mahwah, USA). This process is based on a CT scan and a generated 3D model.

The preoperative plan follows FKP principles to achieve balanced flexion and extension gaps. The distal femoral resection removes 9 mm from the medial condyle (7 mm bone, 2 mm cartilage) and 4–6 mm from the lateral condyle, accounting for bone loss. For the tibial plateau, 8 mm is resected medially (6 mm bone, 2 mm cartilage) and 4–6 mm laterally, depending on wear. The total medial resection is designed to match the implant thickness, ensuring that the thinnest tibial liner remains at 17 mm. Flexion and extension gaps should remain within 1.5 mm of each other, with final gaps in full extension and 90° flexion not exceeding 2 mm from implant thickness (e.g., ≤19.5 mm for a 17.5 mm implant). Adjustments are made intraoperatively to optimize balance and restore anatomy.

### Step 2: Surgical approach

A lateral skin incision is made, followed by a lateral parapatellar approach with a two-layer capsulotomy, ensuring a 1 cm overlap for secure final closure. The Hoffa fat pad is carefully dissected from the underside of the patellar tendon, releasing its medial attachment while preserving its lateral retinacular attachment and blood supply. Retaining the Hoffa fat pad can aid in closing the lower retinacular incision, especially after significant valgus correction. Soft tissue release is performed as needed based on case-specific requirements. The patella is medially subluxated to improve joint access.

### Step 3: Knee evaluation with an image-based robotic system

**Figure 2:**
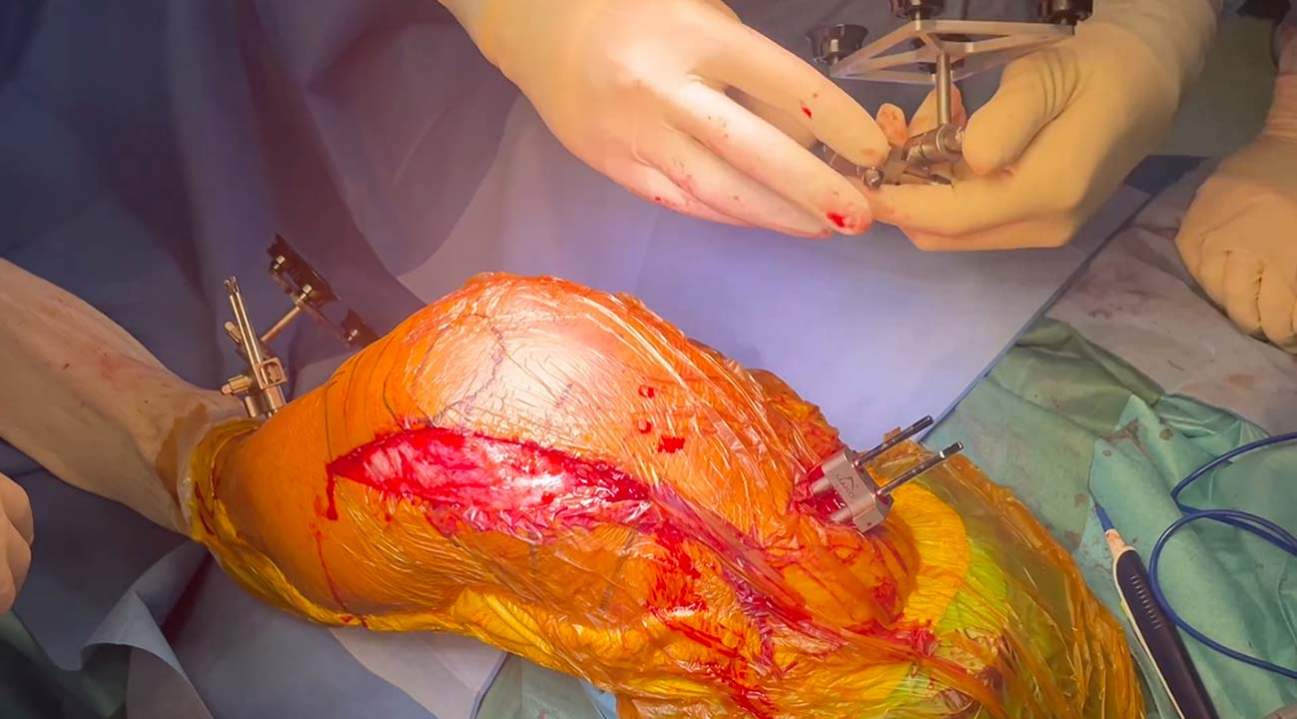
Placement of the femoral pins via two anteromedial stab incisions at the mid-diaphysis of the femur.

For the robotic phase, femoral pins are inserted via two anteromedial stab incisions at the mid-diaphysis of the femur, while tibial pins are placed through two anteromedial stab incisions in the distal diaphysis. The femoral pin placement, which differs from the traditional approach, is necessary to avoid impingement with the cutting saw and to allow extensor mechanism reduction during the procedure (Fig. 2). Arrays are positioned, and the hip center of rotation, bony landmarks, and cartilage thickness are recorded and matched with the preoperative CT model.

Intraoperative coronal alignment assessments are performed at full extension, 90° flexion, and maximum flexion. Laxity evaluations are conducted at approximately 10° of flexion to prevent posterior capsule tension and at 90° of flexion in both compartments, using manual valgus and varus stress in extension and specific spoons in flexion. These intraoperative assessments allow for a real-time comparison between the planned bone cuts and the observed laxity in each compartment.

### Step 4: Intraoperative planning and femoral and tibial cuts

**Figure 3:**
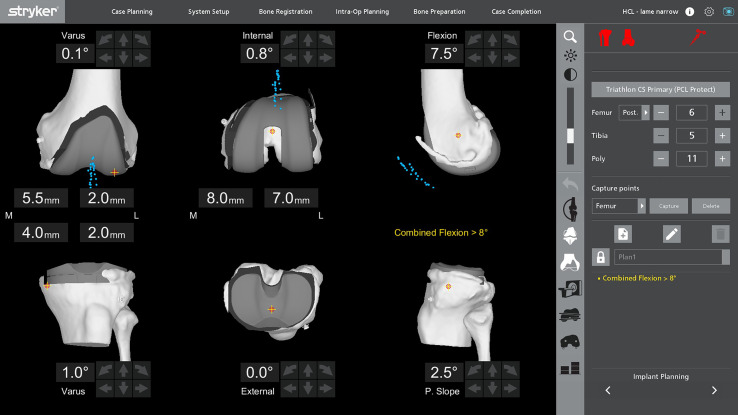
Intraoperative screenshot of TKA positioning using Mako^®^ (Stryker^®^, Mahwah, USA). The implant positioning was performed following functional alignment, also known as functional knee positioning.

To optimize knee balance, femoral and tibial cuts are adjusted to achieve 0 mm gaps in extension and flexion for both lateral and medial compartments in posterior-stabilized (PS) implants. For cruciate-retaining (CR) implants with a cruciate-substituting (CS) liner, the target gaps are up to 1.5 mm in flexion for the lateral compartment and 1 mm for the medial compartment. The cut thickness is maintained between 1.5 and 10 mm. A strict evaluation is conducted to ensure trochlear compatibility of the implant with the native anatomy. Additionally, patellar tracking is evaluated using a robotic tool (Fig. 3).

Once the planning is confirmed, a robotic arm is utilized to perform precise femoral and tibial cuts. The femoral cuts remain unchanged from the medial approach technique; however, careful tibial exposure is required. A Hohmann retractor should be placed medially to protect the patellar tendon, while an extended boundary workflow facilitates the tibial cut, beginning from the anterolateral aspect to avoid patellar tendon damage.

### Step 5: Final evaluations with the trial components

With the trial components, several key evaluations are performed to ensure proper implant positioning. The final coronal alignment is reassessed at full extension, 90° flexion, and maximum flexion, targeting a “safe zone” between 177° and 183°. Residual gaps and patellar tracking are also re-evaluated.

Special attention should be given to femoral medio-lateral positioning, as the lateral approach may increase the risk of misalignment for surgeons more familiar with the medial approach. It is also crucial to ensure clear visualization of the posteromedial tibial plateau to prevent excessive lateral rotation of the tibial component, a critical factor in this technique.

Additionally, proper tibial implant coverage must be confirmed. Tibial rotation is determined using a combination of the floating method, Akagi’s line, and robotic alignment with the preoperative plan.

Patellar resurfacing is performed as needed, considering patient characteristics and surgeon preference.

### Step 6: Placement of the final implant and capsular closure

The final implants, either CS or PS fixed bearing designs (Triathlon^®^, Stryker^®^, Mahwah, USA) are positioned. In this approach, closure is a crucial step. The joint is closed with the knee flexed at 90°. The angular position is first secured, followed by proximal closure. The overlapping two layers of the capsule proximally and any remaining fat pad distally are used to reinforce the joint closure, ensuring a tight seal for the knee. Finally, any excess fat pad is removed.

**Figure 4:**
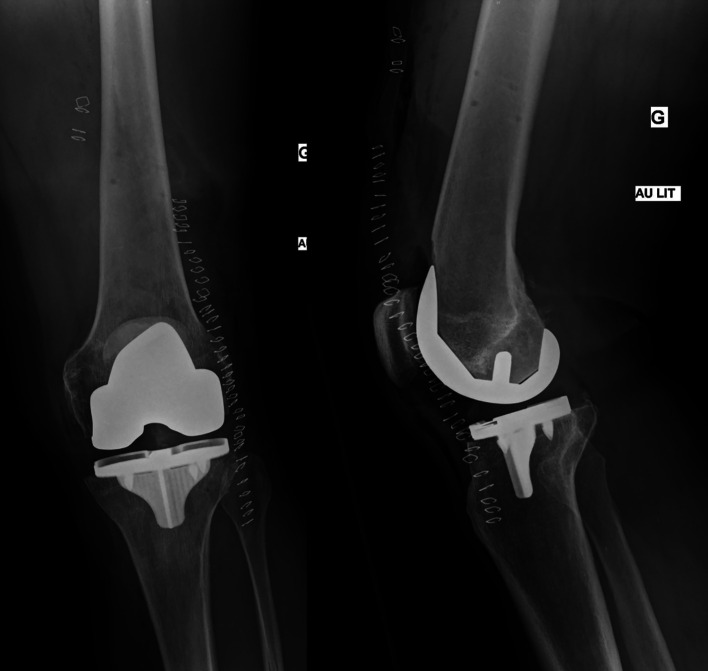
Postoperative X-rays after the placement of the final implant with a cementless femoral and tibial component (Triathlon, Stryker, MI, USA).

Figure 4 shows the preoperative X-rays.

## Discussion

This article describes and illustrates the FKP technique with a lateral approach to the knee in patients with a valgus morphotype. TKA in valgus knees has traditionally been considered more challenging than TKA for varus deformities, not only due to the presence of multiple phenotypes but also because of the need for adequate access and joint visualization [8, 9].

The lateral approach in TKA is primarily indicated for valgus deformities, which present unique anatomical challenges such as lateral femoral condyle hypoplasia, femorotibial malrotation, and patellar subluxation [3, 4]. Soft tissue deficiencies can make prosthetic coverage and joint sealing difficult. Correcting valgus contractures typically requires sequential soft tissue releases, including the lateral capsule, iliotibial band (ITB), vastus lateralis tendon, and occasionally the lateral collateral ligament (LCL), popliteus, and lateral gastrocnemius [10]. This approach has few contraindications but should be avoided if there is a previous scar within 6–8 cm of the incision site due to skin viability concerns [11].

The choice between a lateral or medial approach may positively impact surgical outcomes, although meta-analyses in the literature show conflicting results. While some systematic reviews indicate that both approaches result in similar postoperative Knee Society Scores (KSS), other analyses suggest that the lateral approach is associated with higher KSS knee and function scores [1, 4, 12]. However, both approaches showed comparable results in surgical time and valgus deformity correction, as well as in postoperative outcomes such as range of motion and complication rates [1, 4, 12].

An individualized approach may play a role in addressing this complexity. FKP offers a promising soft tissue-driven personalized alignment for TKA, making it a potential solution for complex cases. However, careful patient selection is crucial. The integrity of the soft tissue envelope is a key prerequisite for FKP, meaning that patients with rheumatoid arthritis, prior trauma, ligament injuries, or a history of high tibial osteotomy may not be ideal candidates.

Robotic-assisted TKA has been widely recognized as a safe and reliable technique, with an increasing number of systems available [13, 14]. Compared to manual TKA, robotic techniques have demonstrated superior short-term outcomes, particularly in terms of precision and functional recovery, while mid-term results appear comparable [15]. The evolution of these approaches reflects a broader shift toward patient-specific TKA strategies, with growing evidence supporting enhanced functional outcomes, higher patient satisfaction, and improved knee kinematics. Nevertheless, the long-term durability of these techniques remains an area of ongoing research, underscoring the need for further high-quality studies. Additionally, the ability to employ various surgical approaches with robotic technology is crucial for optimizing patient outcomes and adapting to diverse clinical scenarios.

## Conclusions

The integration of the lateral approach with robotic-assisted TKA and FKP represents a promising advancement in the management of valgus knee deformities. The technique is effective and reproducible. Future studies will help refine FKP strategies and optimize patient outcomes in complex knee morphotypes.

## Data Availability

The video is available as supplementary material.

## Supplementary material

*Video 1*. Lateral approach. The video is available at the following https://sicotj.edpsciences.org/10.1051/sicotj/2025017#V1.

## References

[1] Rajnish RK, Srivastava A, Yadav SK, et al. (2024) Comparative analysis of outcomes of lateral versus medial approach in the total knee arthroplasty for valgus deformity: a systematic review and meta-analysis. Indian J Orthop 58, 1323–1338. 39324082 10.1007/s43465-024-01211-6 PMC11420410

[2] Nikolopoulos D, Michos I, Safos G, Safos P (2015) Current surgical strategies for total arthroplasty in valgus knee. World J Orthop 6, 469–482. 26191494 10.5312/wjo.v6.i6.469 PMC4501933

[3] Alsalloum M, Alimy A-R, Hubert J, et al. (2024) The lateral approach for total knee arthroplasty in valgus osteoarthritis provides similar clinical and radiological results compared with the medial approach. Knee Surg Sports Traumatol Arthrosc. 10.1002/ksa.12526. 39474862

[4] Mercurio M, Gasparini G, Galasso O, et al. (2023) Lateral versus medial approach for total knee arthroplasty for valgus knee deformity shows comparable functional outcomes, hip–knee–ankle angle values, and complication rates: a meta-analysis of comparative studies. Arch Orthop Trauma Surg 144, 869. 37864590 10.1007/s00402-023-05088-2 PMC10822808

[5] Shatrov J, Foissey C, Kafelov M, et al. (2023) Functional alignment philosophy in total knee arthroplasty-rationale and technique for the valgus morphotype using an image based robotic platform and individualized planning. J Pers Med 13, 212. 36836446 10.3390/jpm13020212 PMC9961945

[6] Gregori P, Koutserimpas C, De Fazio A, et al. (2025) Functional knee positioning in patients with valgus deformity undergoing image-based robotic total knee arthroplasty: surgical technique. SICOT J 11, 7. 39927688 10.1051/sicotj/2025001 PMC11809196

[7] Batailler C, Lording T, Libert T, et al. (2025) Achieving better clinical outcomes after total knee arthroplasty in knees with valgus deformity: the role of alignment strategies. J Bone Joint Surg Am 107, 152–162. 39591439 10.2106/JBJS.24.00207

[8] Mullaji A, Bhoskar R, Singh A, Haidermota M (2022) Valgus arthritic knees can be classified into nine phenotypes. Knee Surg Sports Traumatol Arthrosc 30, 2895–2904. 34750671 10.1007/s00167-021-06796-1

[9] Rossi SMP, Sangaletti R, Andriollo L, et al. (2024) The use of a modern robotic system for the treatment of severe knee deformities. Technol Health Care 32, 3737–3746. 38251078 10.3233/THC-231261

[10] Gregori P, Koutserimpas C, Giovanoulis V, et al. (2025) Functional alignment in robotic-assisted total knee arthroplasty for valgus deformity achieves safe coronal alignment and excellent short-term outcomes. Knee Surg Sports Traumatol Arthrosc. 10.1002/ksa.12585. PMC12104782 39821487

[11] Cheng W, Li Z, Zhang J, et al. (2021) A lateral parapatellar approach with iliotibial band dissection from the Gerdy tubercle for total knee arthroplasty of the valgus knee. Exp Ther Med 21, 38. 33273968 10.3892/etm.2020.9470 PMC7706401

[12] Xu G, Fu X, Tian P, et al. (2020) The lateral and medial approach in total arthroplasty for valgus knee: a meta-analysis of current literature. J Comp Eff Res 9, 35–44. 31777265 10.2217/cer-2019-0111

[13] Prakash R, Agrawal Y (2023) Robotic technology in total knee arthroplasty. Br J Hosp Med (Lond) 84, 1–9. 10.12968/hmed.2022.0491 37364881

[14] Andriollo L, Benazzo F, Cinelli V, et al. (2024) The use of an imageless robotic system in revision of unicompartmental knee arthroplasty. Knee Surg Sports Traumatol Arthrosc. 10.1002/ksa.12574. PMC12022834 39740128

[15] Kafelov M, Batailler C, Shatrov J, et al. (2023) Functional positioning principles for image-based robotic-assisted TKA achieved a higher Forgotten Joint Score at 1 year compared to conventional TKA with restricted kinematic alignment. Knee Surg Sports Traumatol Arthrosc 31, 5591–5602. 37851026 10.1007/s00167-023-07609-3

